# Tetra-Amelia Syndrome

**DOI:** 10.21699/ajcr.v7i4.459

**Published:** 2016-09-01

**Authors:** Shilpi Gupta, vineeta Chaturvedi, Liladhar Agarwal

**Affiliations:** Department of Pediatric Surgery, Sawai Man Singh (SMS) Medical College and Hospital, Jaipur, India

**Dear Sir,**

A newborn was noticed lying unattended in the hospital campus. It had complete absence of all four limbs, deformed ears, facial cleft, proptosis, hydrocephalus, corneal opacity, absent nipples, anterior abdominal wall defect with exstrophy bladder, absent external genitalia, and sacral spina bifida (Fig.1 A and B). The child died after few hours due to aspiration pneumonitis. These features are part of ‘tetra-amelia syndrome’ (TAS) which is an extremely rare autosomal recessive congenital disorder, characterized by absence of all four limbs and anomalies involving the cranium and face, urogenital system, anorectum, heart, lungs, skeleton, and central nervous system.[1] Mutation in WNT3 gene (Chromosome 17q21) has been linked to TAS. Prenatal molecular genetic testing is possible even before routine prenatal ultrasonography can detect TAS. Infants are often stillborn or die soon after birth. However, it is important to note that even complete absence of all extremities per-se is not incompatible with life. Management and survival is guided by presence and severity of associated anomalies. Such cases obviously need major assistance for most routine activities. This case highlights the social stigma associated with birth of a child with TAS. Parental and family counseling is of utmost importance for acceptance and further genetic testing.

**Figure:**
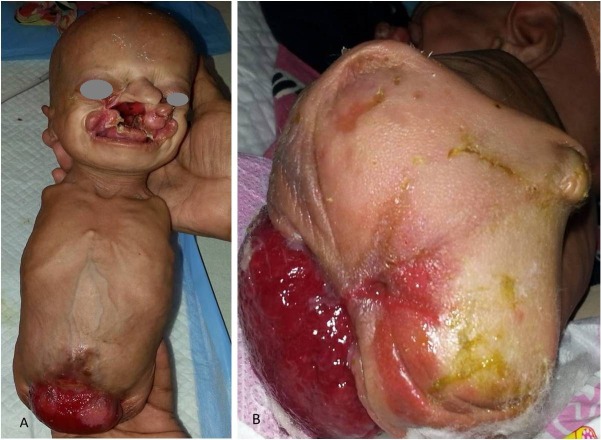
Figure 1 A and B: Showing absence of all the four limbs facial cleft, left corneal opacity, absent nipples, abdominal wall defect, exstrophy bladder and absent genitalia.

## Footnotes

**Source of Support:** Nil

**Conflict of Interest:** None declared

